# Language delay aggregates in toddler siblings of children with autism spectrum disorder

**DOI:** 10.1186/s11689-018-9247-8

**Published:** 2018-10-22

**Authors:** N Marrus, L P Hall, S J Paterson, J T Elison, J J Wolff, M R Swanson, J Parish-Morris, A T Eggebrecht, J R Pruett, H C Hazlett, L Zwaigenbaum, S Dager, A M Estes, R T Schultz, K N Botteron, J Piven, J N Constantino, for the IBIS Network, J Piven, H C Hazlett, C Chappell, S Dager, A Estes, D Shaw, K Botteron, R McKinstry, J Constantino, J Pruett, R T Schultz, S Paterson, L Zwaigenbaum, J Elison, A C Evans, D L Collins, G B Pike, V Fonov, P Kostopoulos, S Das, G Gerig, M Styner, H Gu

**Affiliations:** 1 Department of Psychiatry, Washington University School of Medicine, 660 S. Euclid Ave, Box 8504, St Louis, MO 63110 USA; 2 Department of Psychology, St. Jude Children’s Research Hospital, 262 Danny Thomas Place, Mail Stop 740, Memphis, TN 38105 USA; 3 Department of Psychology, Temple University, 1801 N. Broad St, Philadelphia, PA 19122 USA; 4 Institute of Child Development, University of Minnesota, 51 East River Parkway, Minneapolis, MN 55455 USA; 5 Department of Educational Psychology, University of Minnesota, 56 East River Road, Minneapolis, MN 55455 USA; 6 Department of Psychiatry, University of North Carolina at Chapel Hill, 101 Manning Dr, Chapel Hill, NC 27514 USA; 7 Children’s Hospital of Philadelphia, University of Pennsylvania, Civic Center Blvd, Philadelphia, PA 19104 USA; 8 Mallinckrodt Institute of Radiology, Washington University School of Medicine, 660 S. Euclid Ave, St Louis, MO 63110 USA; 9 Department of Pediatrics, University of Alberta, 1E1 Walter Mackenzie Health Sciences Centre (WMC), 8440 112 St NW, Edmonton, AB T6G 2B7 Canada; 10 Department of Radiology, University of Washington, Seattle, 1410 NE Campus Parkway, Seattle, WA 98195 USA; 11 Department of Speech and Hearing Sciences, University of Washington, Seattle, 1701 NE Columbia Rd, Seattle, WA 98195-7920 USA

**Keywords:** Language, Infant sibling, Endophenotype, Autism spectrum disorder, Development

## Abstract

**Background:**

Language delay is extremely common in children with autism spectrum disorder (ASD), yet it is unclear whether measurable variation in early language is associated with genetic liability for ASD. Assessment of language development in unaffected siblings of children with ASD can inform whether decreased early language ability aggregates with inherited risk for ASD and serves as an ASD endophenotype.

**Methods:**

We implemented two approaches: (1) a meta-analysis of studies comparing language delay, a categorical indicator of language function, and language scores, a continuous metric, in unaffected toddlers at high and low familial risk for ASD, and (2) a parallel analysis of 350 unaffected 24-month-olds in the Infant Brain Imaging Study (IBIS), a prospective study of infants at high and low familial risk for ASD. An advantage of the former was its detection of group differences from pooled data across unique samples; an advantage of the latter was its sensitivity in quantifying early manifestations of language delay while accounting for covariates within a single large sample.

**Results:**

Meta-analysis showed that high-risk siblings without ASD (HR-noASD) were three to four times more likely to exhibit language delay versus low-risk siblings without ASD (LR-noASD) and had lower mean receptive and expressive language scores. Analyses of IBIS data corroborated that language delay, specifically receptive language delay, was more frequent in the HR-noASD (*n* = 235) versus LR-noASD group (*n* = 115). IBIS language scores were continuously and unimodally distributed, with a pathological shift towards decreased language function in HR-noASD siblings. The elevated inherited risk for ASD was associated with lower receptive and expressive language scores when controlling for sociodemographic factors. For receptive but not expressive language, the effect of risk group remained significant even when controlling for nonverbal cognition.

**Conclusions:**

Greater frequency of language delay and a lower distribution of language scores in high-risk, unaffected toddler-aged siblings support decreased early language ability as an endophenotype for ASD, with a more pronounced effect for receptive versus expressive language. Further characterization of language development is warranted to refine genetic investigations of ASD and to elucidate factors influencing the progression of core autistic traits and related symptoms.

**Electronic supplementary material:**

The online version of this article (10.1186/s11689-018-9247-8) contains supplementary material, which is available to authorized users.

## Background

A major challenge in elucidating the biology underlying autism spectrum disorder (ASD) is its genetic heterogeneity. Endophenotypes, heritable characteristics which share genetic liability with a disorder and which are measurable regardless of a disorder’s state or stage (i.e., state-independent) [1, 2], are therefore especially informative for resolving the complex, polygenic genetic architecture of ASD. By definition, endophenotypes demonstrate several criteria involving inheritance among family members with and without the disorder. These criteria include co-segregating, or being inherited more commonly, in affected versus unaffected family members and aggregating, or occurring with increased frequency, in unaffected family members versus the general population, which is at lower genetic risk [1, 2]. The occurrence of these familial patterns in the context of a heritable trait substantiates the relationship between an endophenotype and genetic factors for a given disorder. Because endophenotypes can be inferred to reflect causal pathways of a disorder and can be reliably measured in individuals with and without the disorder [1, 2], they enhance the sensitivity to determine contributory genes and, by extension, the underlying biology.

The common co-occurrence of ASD and persistent language impairments [3], which may include deficits in aspects of structural language, such as vocabulary and grammar, as well as pragmatics, the appropriate use of language, has prompted the long-standing question of whether language deficits represent an endophenotype of ASD [4, 5]. Like ASD, language disorders are heritable [6–9], with evidence of genetic influence from early in development [10, 11]. Further, as expected for an endophenotype, both autistic symptoms and language ability appear to behave as quantitative traits which are heritable across a range of competency encompassing unimpaired and impaired individuals [9, 12–15]. Multiple family studies have investigated the potential role of language function as an ASD endophenotype by examining whether language impairment occurs with increased frequency in families with a history of ASD. Although several of these studies have reported that unaffected family members of individuals with ASD show more language impairment than expected for the general population [16–25], others have failed to find a difference from expected rates in a control population [26–29]. Conversely, other groups have found an increased prevalence of ASD in family members of individuals with a specific language impairment (SLI) [30, 31], but again, this finding has not been universal [27, 32]. Interpretation of the literature is complicated by several factors limiting the comparability across studies, including differing diagnostic criteria for language impairment (e.g., [26] versus [22]); small sample sizes [23, 29]; broad participant age ranges [33], which may mask developmentally sensitive manifestations of language function; and lack of a control group [24] or standardized language assessment [21, 25]. Given this inconsistency, the field has been challenged to arrive at a consensus regarding whether language function operates as an ASD endophenotype.

Related work examining quantitative relationships between autistic traits and language function generally supports overlapping genetic factors, in keeping with a language-related ASD endophenotype. For example, a recent general population twin study found shared genetic influences for early childhood language scores at age 2 years and quantitative measures of autistic traits at school age [34]. In a study measuring autistic traits in unaffected siblings of individuals with ASD [35], higher levels of autistic traits were observed in siblings with a history of language delay, again supporting overlapping genetic influences for ASD and language delay, as well as an amplification of ASD risk with a co-occurring history of language delay. Multiple genetic studies have identified genes, such as contactin-associated protein-like 2 (CNTNAP2) [36–38], and genetic loci, particularly on chromosome 7 [39–43], which are associated with both ASD and specific language impairment. These convergent findings imply that shared genes may lead to disruptions of social and language development, and language delay is frequently observed in ASD [44–46]. A relevant question is therefore whether language delay, which entails altered emergence of foundational language skills (e.g., comprehension and production of words, word combinations, and simple sentences), and which displays some continuity with later language function [10, 47–50], aggregates in unaffected toddler siblings of individuals with ASD. Identification and characterization of an early language endophenotype has implications for enhancing diagnostic sensitivity and risk stratification, clarifying developmental mechanisms, and refining targets for early interventions.

To test whether early language delay is an endophenotype of ASD, we leveraged data from infant sibling studies, developmental family studies designed to identify early predictive risk factors of ASD. In these genetically informative study designs, infant siblings of children with ASD, who are at elevated familial risk for ASD, as well as sibling controls at low familial risk for ASD, undergo standardized behavioral and diagnostic testing. Because these studies generally involve similar ages and assessments, comparable data can be pooled across samples to improve power to detect traits associated with inherited ASD risk. By comparing risk groups, endophenotypes can be identified as features enriched in those at elevated familial risk of ASD. Evaluating differences in language function between high-risk siblings without ASD (HR-noASD siblings) and low-risk siblings without ASD (LR-noASD siblings) affords a particularly stringent test for an endophenotype, since differences between these unaffected groups are not biased by the high comorbidity of language deficits in ASD [44–46] and are attributable to the presence of familial ASD liability. Additionally, this comparison disambiguates the role of inherited ASD risk, which is linked to underlying mechanisms of ASD, from consequences of ASD itself, thereby facilitating investigation of the role of language-related factors, such as sociodemographic variables, nonverbal cognition, and ASD-related social deficits, in the manifestation of an endophenotype.

Given extant literature on HR-noASD infant siblings, we conducted a meta-analysis as a first step to test whether language delay, as well as lower mean language scores, aggregate in HR-noASD toddlers. We focused on the toddler period since broad variation in advancing language abilities at this stage could enhance detection of group-level differences. We hypothesized that if disruptions in early language associate with inherited ASD risk, a greater frequency of language delay and lower mean language scores would be observed in HR-noASD siblings versus LR-noASD siblings. Based on the results of this meta-analysis, we tested whether observed differences could be replicated and extended using data from the Infant Brain Imaging Study (IBIS), a large infant sibling study [51]. The IBIS cohort expanded the sample for meta-analysis while allowing more comprehensive analyses which controlled for sociodemographic factors not universally reported in published studies, examined the distribution of language scores for each risk group, and investigated the relationship between language and nonverbal cognition, as well as language and ASD-related social deficits. We hypothesized that if language delay were an endophenotype, the HR-noASD group in IBIS would show an increased prevalence of language delay versus the LR-noASD group, a downward shift in distributions of language scores, and lower mean language scores versus the LR-noASD group, even when sociodemographic factors were controlled.

## Methods

### Literature review

To review the available literature for evidence of associations between familial ASD risk and decreased language, we searched for published articles both in PubMed, a database with strong representation of clinical literature, and Scopus, a database with broader coverage in the social sciences [52]. The search used the keywords “autism,” “language,” and “sibling” for manuscripts published since 2000, the year the DSM-IV-TR was published [53]. Inclusion criteria were as follows: (1) analysis of a high-risk group of toddlers, here considered children with a mean age of 12–24 months, who had a sibling with ASD but no ASD diagnosis themselves (HR-noASD siblings), (2) analysis of a low-risk group of toddlers who had a typically developing sibling and no ASD diagnosis themselves (LR-noASD siblings), (3) a clinical best estimate diagnosis of ASD for children aged 24 months and up, an early age with evidence for diagnostic stability [54–57], (4) implementation of standardized language assessments from 1 to 2 years of age, as this particular period captures variation in the early emergence of spoken language, and (5) reporting of language scores or standardized criteria for language delay for both HR-noASD and LR-noASD groups. Studies without a diagnostic evaluation of toddlers below age 24 months, when symptoms of ASD are less likely to have stably emerged [58, 59], were retained for consideration in the meta-analysis to promote broader representation of unique study populations, which enhances the generalizability of findings in a meta-analysis [60].

The search identified 216 articles published between January 1, 2000, and May 31, 2017. One hundred nineteen of these involved HR-noASD siblings. Fifty-seven articles reported on toddlers (i.e., children with a mean age of 12–24 months), and 52 of these examined aspects of language development. Twenty-six of the 52 articles satisfied inclusion criteria. Among these 26 articles, 15 articles were eliminated because they shared participants with another study, either due to multiple manuscripts about the same population or due to manuscripts describing a consortium of studies. The remaining 11 articles represented data from all samples in the eliminated articles and were chosen due to having the largest number of children closest to 24 months of age, a common assessment time point expected to exhibit a broader range of spoken language abilities than younger ages. Where language data were available from multiple measures within a study, continuous scores from the Mullen Scales of Early Learning (MSEL) [61], the most frequent assessment encountered, were selected to enhance comparability across studies as well as IBIS, which featured MSEL data. One study [62], which reported age equivalent scores on the MSEL but did not control for age, was excluded to avoid confounding age differences with differences in language ability. To promote uniformity in the meta-analysis of continuous language scores, one additional study population was excluded [63, 64], as it used the MacArthur-Bates Communicative Development Inventories [65], a parent-report measure.

**Fig. 1:**
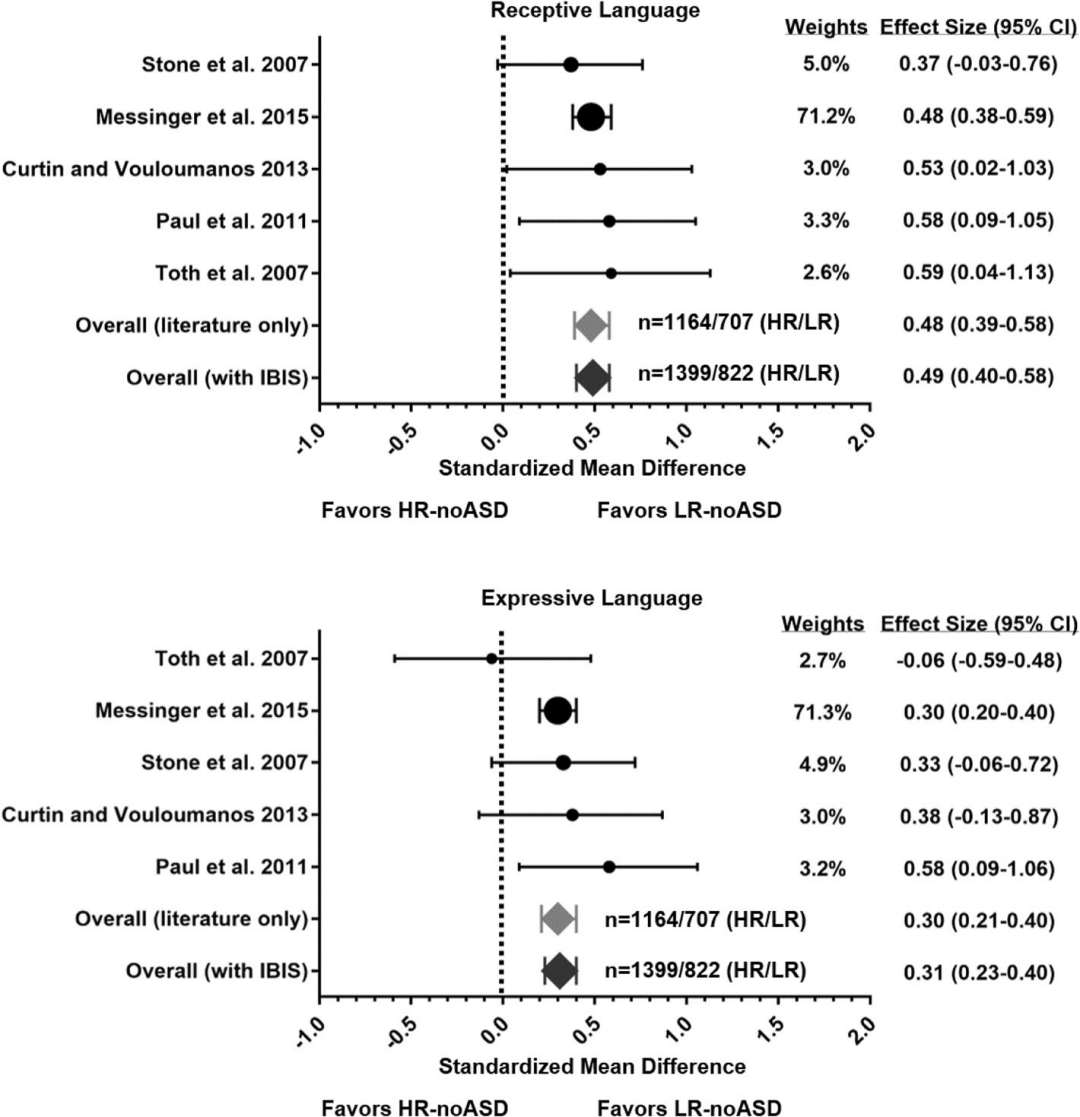
Meta-analysis of language scores in high-risk siblings without ASD. Forest plots display the results of the meta-analysis examining differences in receptive and expressive language scores between low-risk siblings without ASD (LR-noASD) and high-risk siblings without ASD (HR-noASD). Circle sizes illustrate each study’s weighted impact when including IBIS data, with values for weights and effect sizes listed on the right. Error bars represent 95% confidence intervals (CI). Summary weighted effect sizes for published studies only, indicated as “Overall (literature only),” are shown as a light gray diamond; the dark gray diamonds show the result including IBIS data. Numbers of subjects in HR-noASD (HR) and LR-noASD (LR) groups are adjacent to these diamond markers. Both summary effect sizes indicate that receptive and expressive language scores are significantly lower in HR-noASD siblings. The effect size is moderate for receptive language and small for expressive language

A large study from the Baby Sibling Research Consortium (BSRC) [66], which was included in the main meta-analysis presented in Fig. 1, differed from other studies in that it reported continuous language scores as estimated marginal means (rather than *T*-scores) based on a model testing effects of sex, age, language subscale, and diagnostic group on language outcome. This study shared subjects with some smaller studies containing appropriate continuous language data that were excluded from the main meta-analysis of continuous scores [45, 67, 68]. For purposes of comparison, supplemental analyses repeat the meta-analysis using these smaller studies instead of this large BSRC study and show consistent results (Additional file 1: Table S1 and Figure S1). Note that one of these smaller studies, Mitchell et al. [45], contained categorical data on language delay, which were included in that segment of the meta-analysis reported in the main text (Table 1).

**Table 1:** Study characteristics of publications in meta-analysis

Publication	HR/LR	Mean age (mo.)	Language delay criteria	Clinical best estimate measures	Language delay (HR versus LR)	MSEL receptive language	MSEL expressive language
Landa and Garrett-Mayer [72]	38/25	24	≤ 1 SD mean: M-CDI or PLS	ADOS	OR = 3.57 (.70–18.16) *χ*^2^ = 1.60, *p* = .21	n/a	n/a
Iverson and Wozniak [115]	14/18	18	≤ 5th percentile: M-CDI words produced	n/a	**OR = 37.00 (1.87–732.72)** ***χ***^**2**^ **= 8.78,** ***p*** **= .003**	n/a	n/a
Gamliel et al. [116]	38/38	24	≤ 2 SD mean: BSID or RDLS	ADOS-G ADI-R	**OR = 7.33 (1.50–35.86)** ***χ***^**2**^ **= 5.94,** ***p*** **= .015**	n/a	n/a
Mitchell et al. [45]	91/52	24	≤ 1.5 SD mean: MSEL or PLS	ADOS DSM-IV-TR	OR = 1.65 (.50–5.47) *χ*^2^ = .29, *p* = .59	n/a	n/a
Toth et al. [117]	42/20	21	n/a	ADOS ADI-R DSM-IV-TR	n/a	**54.50 (6.40)** **46.55 (15.60)**	48.45 (6.65) 49.07(12.24)
Stone et al. [118]	64/42	16	n/a	n/a	n/a	48.2 (10.8) 43.8 (12.6)	48.7 (10.5) 45.1 (11.1)
Paul et al. [119]	38/31	12	n/a	n/a	n/a	**46.1 (7.2) 41.1 (9.7)**	**47.4 (12.0)** **40.8 (10.7)**
Curtin and Vouloumanos [120]	31/31	12	n/a	n/a	n/a	**45.97 (7.11)** **42.16 (7.14)**	53.93 (10.47) 49.87 (11.04)
Messinger et al. [66]	989/583	24	n/a	ADOS MSEL	n/a	**27.28 (5.12)** **24.63 (5.67)**	**25.78 (5.29)** **24.11 (5.78)**

Except where stated, language delay was defined as either receptive or expressive language delay. Odds ratios (ORs) are listed with 95% confidence intervals in parentheses. With the exception of Messinger et al., 2015, which used age equivalent scores at 24 months of age, language scores consisted of *T*-scores on the Mullen Scales of Early Learning (MSEL), with low-risk siblings without autism spectrum disorder (ASD) listed first and high-risk siblings without ASD listed second. Bolded values indicate significant risk group differences at *p* < .05. *mo.* months, *HR* high-risk siblings without ASD, *LR* low-risk siblings without ASD, *M-CDI* MacArthur Communicative Development Inventory, *BSID* Bayley Scales of Infant Development, *RDLS* Reynell Developmental Language Scales, *PLS* Preschool Language Scale, *ADOS* Autism Diagnostic Observation Schedule, *ADOS-G* Autism Diagnostic Observation Schedule-General, *ADI-R* Autism Diagnostic Interview-Revised, *DSM-IV-TR* Diagnostic and Statistical Manual of Psychiatric Disorders, 4th edition, text revision

Lastly, meta-analytic findings from the literature review are first presented without incorporating IBIS data, since these results motivated subsequent analyses in IBIS to test for replicability and the impact of covariates on group differences.

### IBIS sample

The Infant Brain Imaging Study (IBIS) is a longitudinal multisite study of infants at familial risk of ASD by virtue of having a sibling with a diagnosis of ASD, verified by medical records and the Autism Diagnostic Interview-Revised [69]. IBIS also contains a low-risk comparison group of infant siblings, who have no first-degree family members meeting screening criteria for ASD or intellectual disability [51]. Rates of ASD diagnosis in IBIS [51, 70] have been shown to be similar to other infant sibling studies [71]. Participants in the first wave of IBIS, whose data were used in these analyses, were behaviorally assessed and completed magnetic resonance imaging (MRI) during natural sleep at ages 6, 12, and 24 months at the following study sites: the University of North Carolina, the University of Washington, The Children’s Hospital of Philadelphia, and Washington University in St. Louis. The Montreal Neurological Institute served as the data coordination center. Exclusion criteria included: (1) diagnosis or physical signs of known genetic conditions or syndromes, (2) significant medical or neurological conditions or sensory impairments, (3) birth weight < 2000 g and/or gestational age < 36 weeks, (4) significant perinatal adversity and/or exposure in utero to neurotoxins, (5) contraindication for MRI, (6) predominant home language other than English, (7) first degree relative with psychosis, schizophrenia, bipolar disorder, and (8) adopted children or half-siblings of the proband. The majority of analyses presented here involve HR-noASD siblings and LR-noASD siblings. High-risk siblings with ASD were included in a single sub-analysis testing differences between HR-noASD siblings and high-risk siblings with ASD. Informed consent approved by each site’s Human Subjects Review Board was obtained for all families.

### Measures

#### Mullen Scales of Early Learning (MSEL)

The MSEL is a standardized direct assessment of cognitive development normed for ages from birth to 68 months [61]. Subscales include receptive and expressive language, as well as visual receptive and motor skills. Mullen *T*-scores, based on standardized norms accounting for age, were used to index levels of receptive and expressive language function. Language delay was defined as a *T*-score ≤ 35, or 1.5 standard deviations below the mean standard score, in accordance with common practice [70, 72], on either receptive or expressive language subscales. A nonverbal composite score was created by averaging the *T*-scores for visual reception and fine motor subscales.

#### Autism Diagnostic Observation Schedule (ADOS)

The ADOS [73] is a semi-structured play assessment of characteristic features of ASD in the domains of communication, social interaction, play skills, and restricted interests/repetitive behavior. ADOS module 1 or 2, designed for different levels of language development, was administered to all subjects at 24 months by certified evaluators who were research reliable across all four sites [51]. Ratings were based on the severity and number of ASD symptoms demonstrated during the assessment, and scores were calculated using empirically derived, conventional scoring algorithms comprised of items identified as strong contributors to variance in prior factor analysis of the ADOS [74]. To maximize the range of detectable variation in children without ASD, the summed item-level scores from the ADOS social affect scoring algorithm, based on a previously identified ADOS social affect factor measuring ASD-related social deficits [74], were used as an index of social performance. Higher social affect scores corresponded to an increased burden of ASD-related deficits. In the supplement, results for analyses using calibrated severity scores for social affect [75] are presented with similar findings (Additional file 1: Table S2).

#### DSM-IV-TR checklist

Diagnoses of ASD were made using a clinical best estimate diagnosis derived from the IBIS behavioral battery and observations during in-person assessment, including the ADOS [51]. Testing, video, and interview data were reviewed by a second experienced clinician to confirm that criteria for an ASD [(autism or Pervasive Developmental Disorder not otherwise specified (NOS)] were met using the DSM-IV-TR checklist at 24 months [53].

### Statistical analyses

The Mantel-Haenszel test, a meta-analytic technique for categorical data [76], was used to evaluate proportions of language delay in HR-noASD versus LR-noASD siblings. To compare language scores in these risk groups across studies, a meta-analysis was performed using a random effects model with inverse variance weighting [77]. First, effect sizes of the differences in language scores between LR-noASD and HR-noASD groups were calculated for each study. Study-specific inverse variance weights, which accounted for sample size and standard error, were then derived, with larger sample size and lower standard errors corresponding to greater inverse variance weight. To standardize the impact of each study in the meta-analysis, each study’s effect size was multiplied by its inverse variance weight. These products were summed and divided by the sum of inverse variant weights for all studies to determine a summary effect size or standardized mean difference. A random effects model was conservatively chosen to account for variance between and within studies, although cross-study heterogeneity was not significant based on the Cochran *Q* statistic [77] (receptive language *Q* = 0.75, df = 4, *p* = .95; expressive language *Q* = 3.48, df = 4, *p* = .48). Age and sociodemographic variables (the latter of which were not uniformly available across studies) were not tested as covariates in these models due to the modest number of studies (< 10), which constrains the ability to accurately estimate the impact of potential moderators through meta-regression [60, 78].

Within the IBIS dataset, differences in participant characteristics between HR-noASD and LR-noASD groups at age 24 months (and in one sub-analysis, between HR-noASD siblings and high-risk siblings with ASD) were examined using *t* tests or *χ*^2^ tests where appropriate for continuous or categorical variables. Children with significant generalized cognitive delay, indicated by a nonverbal developmental composite score ≥ 2 standard deviations below the mean (a level in the bottom 5% of the population) were removed from analyses (HR-noASD *n* = 1; LR-noASD *n* = 1). Binary logistic regression, with ASD-risk status (HR-noASD versus LR-noASD) as the independent variable, was used to test for differences in the presence of language delay (categorized as having or not having language delay), the dependent variable, while controlling for the sociodemographic factors of sex, maternal education (categorized according to those with and without a college degree), income (categorized as greater or less than $75,000 per year), and race (categorized as Caucasian or not Caucasian), as these variables have generally been found to be associated with early language development [79–83]. Sociodemographic factors were entered prior to ASD risk status in these models. Hierarchical linear regressions, with language scores as the dependent variable, tested the influence of ASD risk status, the independent variable, on language ability when controlling for sociodemographic factors, as described above. Additional hierarchical linear regressions examined contributions of nonverbal cognition (nonverbal composite score) and social performance (ADOS social affect score) to variation in language scores. Correlation values for language scores with other behavioral scores were Fisher *z*-transformed to test for significant differences between HR-noASD and LR-noASD groups.

## Results

### Meta-analysis: review of the literature for associations between ASD risk and early language

Our literature review identified nine infant sibling studies with standardized language data in HR-noASD and LR-noASD siblings (Table 1). Four of these studies contained categorical data on the presence of language delay (see Table 1 for individual study criteria), for a total of 181 HR-noASD siblings and 133 LR-noASD siblings. Although all four studies displayed odds ratios consistent with greater language delay in HR-noASD siblings, many of the samples were small, and only two studies showed statistically significant differences in odds ratios between the two risk groups. The Mantel-Haenszel test, which allowed pooling of participants across these studies, showed that HR-noASD siblings were 4.17 (95% CI 1.74–9.99) times more likely to experience language delay than LR-noASD siblings [*χ*^2^_MH_(1) = 14.62, *p* < .001; LR-noASD 6.0% language delay; HR-noASD 21.0% language delay].

For five of the studies (Table 1), comparison of early language ability between HR-noASD and LR-noASD siblings was possible based on continuous scores from the MSEL. These scores provided enhanced sensitivity relative to categorical data for examining risk group differences in receptive and expressive language. Across studies, mean language scores for both groups (Table 1) fell within a normative range (within 1 SD, 10 points, of a mean standard MSEL T-score of 50), although scores were generally lower for the high-risk group. A meta-analysis (Fig. 1) using weighted effect sizes for studies including 1164 HR-noASD siblings and 707 LR-noASD siblings indicated significantly higher receptive language for LR-noASD siblings, with a standardized mean difference (i.e., summary effect size) of 0.48 (95% confidence interval 0.39–0.58). For expressive language, a standardized mean difference of 0.30 (95% confidence interval 0.21–0.40) also indicated significantly higher scores for LR-noASD siblings. Similar effect sizes were observed in a secondary meta-analysis substituting the larger BSRC study with smaller studies sharing some of the same subjects (see Additional file 1: Figure S1 and Table S1). Meta-analysis of the available literature thus suggests that around the age of identification of core ASD symptoms, HR-noASD siblings exhibit more frequent language delay and lower receptive and expressive language scores than low-risk counterparts.

### The Infant Brain Imaging Study (IBIS): testing for replication of language differences in HR-noASD siblings

While the meta-analysis provides evidence for the aggregation of decreased early language skills in unaffected high-risk siblings, the diversity of participant ages and assessments could inflate variability in language measurements, potentially leading to underestimation of differences between risk groups. Additionally, studies with participants under age 24 months, below the usual age of assessment for ASD, included children who could later be diagnosed with ASD. Therefore, we investigated whether a similar result would be observed in the Infant Brain Imaging Study (IBIS), a large infant sibling study sample. IBIS data allowed the analysis of covariates not uniformly available from studies in the meta-analysis as well as augmentation of the meta-analysis. We analyzed children without ASD at 24 months of age, an age anticipated to capture a wider range of measurable variation in language than younger ages (e.g., 12 months, when children are in the process of learning single words). Sample characteristics (*n* = 350) are shown in Table 2. HR-noASD (*n* = 235) and LR-noASD (*n* = 115) did not significantly differ in age, sex, income, or race and showed similar means and standard deviations for the ADOS social affect score. Significant differences between risk groups were observed in maternal education, a nonverbal developmental composite score, and receptive and expressive language scores. The high-risk group showed lower MSEL scores, a higher prevalence of language delay, and a lower percentage of mothers with college or graduate degrees. Comparison of IBIS HR-noASD siblings to high-risk siblings with ASD revealed lower language function in the ASD-affected group, confirming that the IBIS sample is appropriately representative to test a candidate endophenotype (Additional file 1: Supplemental Results).

**Table 2:** Participant characteristics of Infant Brain Imaging Study sample

	LR-noASD (*n* = 115)	HR-noASD (*n* = 235)	Statistics
Sex (*n* male)	69 (60%)	133 (56.6%)	*χ*^2^(1) = .37, *p* = .55
Age (months)	24.61 (.79)	24.69 (.76)	*t*(1) = −.93, *p* = .35
Income [*n* < 75 K (%)]	44 (40.4%)	94 (42.2%)	*χ*^2^(1) = .096, *p* = .76
Race [*n* (% Caucasian)]	100 (87.0%)	205 (87.2%)	*χ*^2^(1) = .005, *p* = .94
Maternal education [*n* (% college degree or above)]	99 (86.1%)	160 (68.1%)	***χ***^**2**^**(1) = 13.01,** ***p*** **< .001**
MSEL nonverbal developmental composite	56.83 (8.54)	51.87 (8.45)	***t*(348) = 5.14,** ***p*** **< .001**
MSEL receptive language score (*T*-score)	56.98 (8.71)	51.79 (10.43)	***t*(348) = 4.61,** ***p*** **< .001**
MSEL expressive language score (*T*-score)	53.23 (10.16)	49.13 (11.29)	***t*(348) = 3.30,** ***p*** **= .001**
Language delay (receptive or expressive) [*n* (%)]	5 (4.3%)	32 (13.6%)	***χ***^**2**^**(1) = 7.02,** ***p*** **= .008**
ADOS social affect score	2.11 (2.36)	2.34 (2.49)	*t*(344) = −.83, *p* = .41

The statistics column shows results of testing for differences in low-risk siblings without autism spectrum disorder (LR-noASD) and high-risk siblings without ASD (HR-noASD siblings). Significant differences are bolded. *MSEL* Mullen Scales of Early Learning, *ADOS* Autism Diagnostic Observation Schedule. The mean *T*-score for the MSEL in the general population is 50, with a standard deviation of 10. Language delay is defined as MSEL receptive or expressive scores ≤ 1.5 standard deviations below the mean

### Comparison of language delay in HR-noASD and LR-noASD siblings in IBIS

To account for sociodemographic factors associated with language outcomes, including sex, maternal education, and income, we performed a binary logistic regression comparing the prevalence of language delay in the LR-noASD and HR-noASD siblings in IBIS. Log of the odds of language delay (coded for as the presence or absence of receptive or expressive language delay) served as the dependent variable. ASD risk group was entered after controlling for sociodemographic variables. The model was significant (*χ*^2^(5) = 13.35, *p* = .02) and showed appropriate goodness of fit (Hosmer’s and Lemeshow’s test *χ*^2^(8) = 3.32, *p* = .91). ASD risk status was a significant contributor to the model (*χ*^2^_Wald_(1) = 5.21, *p* = .022) and accounted for 3.7% of the variance in language delay status, with HR-noASD being 3.18 times (95% CI = 1.18–8.59) more likely than LR-noASD to have a language delay. Sex, maternal education, income, and race were not significant contributors.

To test whether receptive and/or expressive language individually contributed to this effect, analyses were repeated separately for receptive and expressive language delay. The model for receptive language delay was also significant (*χ*^2^(5) = 20.32, *p* = .001) and showed good fit (Hosmer’s and Lemeshow’s test *χ*^2^(7) = 3.24, *p* = .86). Risk status accounted for 5.7% of the variance in receptive language, and HR-noASD siblings were 5.82 times (95% CI 1.30–26.05) more likely to have receptive language delay (*χ*^2^_Wald_(1) = 5.30, *p* = .021). The model for expressive language delay was not significant (*χ*^2^(5) = 6.71, *p* = .24).

### Integration of IBIS data in the meta-analysis of language delay

Inclusion of IBIS percentages for language delay (receptive and/or expressive) in the meta-analysis increased the precision of the findings, with HR-noASD siblings (*n* = 416) being 3.87 times (95% CI 2.04–7.33) more likely to have a language delay than LR-noASD siblings (*n* = 248; *χ*^2^_MH_(1) = 21.32, *p* < .001; HR-noASD 17.0% language delay; LR-noASD 5.2% language delay). When excluding the study of children under age 24 months without diagnostic data [83], the odds ratio was similar [3.29 (95% CI 1.40–7.69), *χ*^2^_MH_(1) = 14.58, *p* < .001; HR-noASD *n* = 402, 15.7% language delay; LR-noASD *n* = 230, 5.7% language delay]. Integration of IBIS with the literature confirms that language delay does aggregate in unaffected, toddler-aged, high-risk siblings.

### Comparison of language scores in HR-noASD and LR-noASD siblings in IBIS

**Fig. 2:**
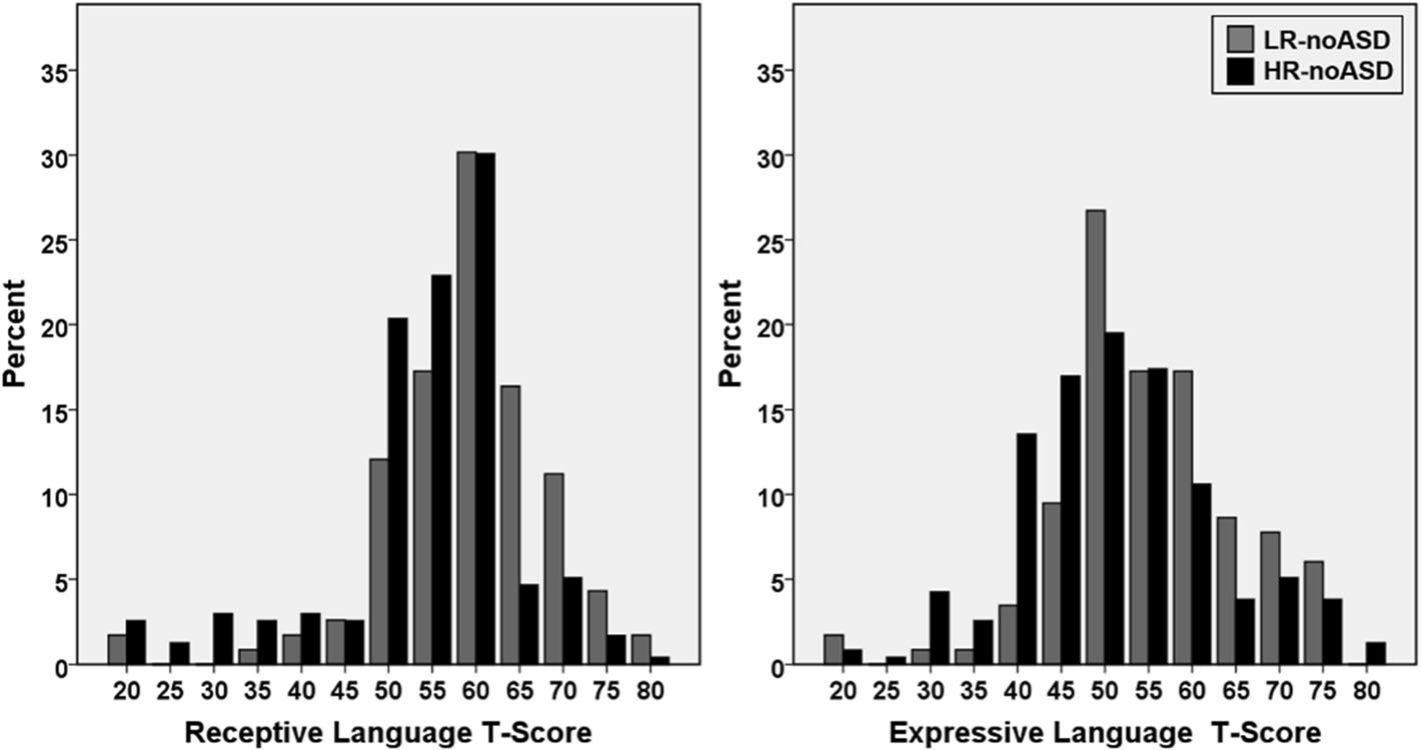
Language scores at 24 months of age in IBIS infant siblings. Histograms display a wide distribution of *T*-scores for Mullen expressive and receptive language scores in the Infant Brain Imaging Study among both the low-risk siblings without ASD (LR-noASD) and high-risk siblings without ASD (HR-noASD). HR-noASD siblings (black bars) generally show a larger percentage of individuals in bins for lower scores, whereas LR-noASD sibling (gray bars) show a larger percentage of individuals in bins with higher scores, signifying a pathological shift in the distribution for the high-risk group

Findings from the meta-analysis do not distinguish whether observed differences in ASD risk groups are attributable to a subgroup of HR-noASD siblings with language impairment or a shift of continuous score distributions towards decreased language function across all HR-noASD siblings. Such a pathological shift would be expected for an endophenotype encompassing the full range of language function, with language delay representing a pathological extreme. Therefore, we next examined the language score distributions for high- and low-risk siblings in IBIS without ASD. Histograms of *T*-scores on the MSEL receptive and expressive language subscales demonstrated a continuous, unimodal distribution for both groups (Fig. 2). Visual inspection of binned scores showed that HR-noASD siblings had a smaller proportion of high scores than LR-noASD siblings and a larger proportion of low scores, corresponding to a pathological shift in score distributions for the HR-ASD siblings. Comparison of proportions for the HR-noASD versus LR-noASD siblings in the top and bottom thirds of the sample was consistent with this downward shift in scores (HR-noASD versus LR-noASD in the top third—receptive 26.4% versus 47.8%, *χ*^2^(1) = 5.71, *p* = 0.017; expressive 26.0% versus 48.7%, *χ*^2^(1) = 6.41, *p* = 0.011; in the bottom third—receptive 39.6% versus 20.9%, *χ*^2^(1) = 2.88, *p* = 0.090; expressive 42.1% versus 15.7%, *χ*^2^(1) = 4.47, *p* = 0.035). Additionally, HR-noASD siblings demonstrated lower mean receptive and expressive language scores than LR-noASD siblings (Table 2) at effect sizes similar to those observed in the meta-analysis (receptive Cohen’s *d* = .54 and expressive Cohen’s *d* = .38).

We next implemented a hierarchical linear regression to test whether language scores, the dependent variable, were predicted by ASD risk status, the independent variable. These models controlled for sociodemographic variables, which were entered first. Receptive and expressive language were analyzed separately, given the distinct effect sizes for these two language domains in the meta-analysis. In the case of receptive language (Table 3), ASD risk status, sex, and income were significant contributors to the model (*F*(5,331) = 10.45, *p* < .001), which accounted for 12.5% of the variance (adjusted *R*^2^) in receptive language scores, with risk status contributing 4.4% of the variance.

**Table 3:** Receptive and expressive language models

	Unstandardized coefficients		Standardized coefficients	*T*	Sig.	95.0% confidence interval for B	
	B	Std. error	Beta			Lower	Upper
**Receptive language model**							
(Constant)	51.19	2.75		18.65	< .001	45.79	56.59
**Sex**	3.57	1.05	.18	3.39	.001	1.50	5.65
Maternal education	2.08	1.27	.092	1.63	.103	− .43	4.59
**Income**	3.27	1.13	.16	2.89	.004	1.05	5.49
Race	− 2.83	1.55	− .094	− 1.82	.069	− 5.88	.23
**ASD risk status**	− 4.69	1.13	− .22	− 4.15	.000	− 6.91	− 2.47
**Expressive language model**							
(Constant)	43.13	3.05		14.13	< .001	37.12	49.13
**Sex**	3.50	1.17	.16	2.98	.003	1.19	5.80
**Maternal education**	3.72	1.42	.15	2.63	.009	.94	6.51
**Income**	2.55	1.26	.11	2.02	.044	.072	5.02
Race	− .043	1.73	− .0010	− .025	.98	− 3.44	3.35
**ASD risk status**	− 3.19	1.26	− .14	− 2.54	.011	− 5.66	− .72

In these models, language (either receptive or expressive) is the dependent variable. ASD-risk status [0 = low-risk sibling without ASD, 1 = high-risk sibling without ASD] is the independent variable. Covariates of sex (0 = male, 1 = female), maternal education (0 = no college, 1 = college or greater), income (0 = <$75,000/year and 1 = ≥$75,000/year), and race (0 = Caucasian, 1 = non-Caucasian) are entered first into the model, followed by risk status. Bolded variables demonstrate a significant relationship with language. For receptive language, sex, income, and ASD risk are significant, with females and higher family incomes associated with higher scores. ASD risk status shows the greatest impact, and greater ASD risk is associated with lower language scores. For expressive language, all variables except race are significant and show similar relative impact. Female sex, higher maternal education, and higher family income are associated with higher scores, while greater ASD risk is associated with lower expressive language scores. *ASD* autism spectrum disorder, *std.* standard, *sig.* significance

As with receptive language, the effect of ASD risk status was significant for expressive language when controlling for sociodemographic variables (Table 3). The total model explained a similar amount of the variance in expressive language, 8.3%, [*F*(5,331) = 6.96, *p* < .001], with risk status accounting for approximately 1.6% of that variance. Thus, in keeping with results for language delay, familial risk of ASD was associated with lower language scores even when controlling for variation attributable to these sociodemographic factors.

### Integration of IBIS data in the meta-analysis of quantitative language scores

Inclusion of the IBIS sample in the meta-analysis of MSEL language scores increased the precision of summary effect sizes for both receptive and expressive language [receptive 0.49 (95% CI 0.40–0.58); expressive 0.31 (95% CI 0.23–0.40)], based on a total of 1399 HR-noASD and 822 LR-noASD siblings (Fig. 1). Removing the studies of toddlers under 24 months without diagnostic data (86–88) yielded similar values [receptive language 0.49 (95% confidence interval 0.40–0.58); expressive language 0.30 (95% confidence interval 0.21–0.40); HR-noASD *n* = 1266; LR-noASD *n* = 718].

### Examination of the specificity of a language endophenotype in IBIS

The above findings demonstrate an association of familial ASD risk with decreased early language function, consistent with a language endophenotype of ASD. To investigate whether ASD risk status was related to variation specific to language, or aspects of language associated with nonverbal cognitive ability, a known predictor of language [84–86], we next evaluated the effect of ASD risk status when nonverbal cognitive ability was included in the model. The MSEL nonverbal composite score was introduced as a third step in the models. In the case of receptive language, the model was significant (*F*(6,331) = 27.10, *p* < .001) and accounted for 32.1% of the variance (adjusted *R*^2^), with nonverbal cognition adding 19.6% variance. Even with the inclusion of the nonverbal score, the effect of ASD risk status on receptive language remained significant (beta = − 2.09*;* SE = 1.03 *p* = .044). In contrast to receptive language, the effect of risk status for expressive language was no longer significant (beta = −.44; SE = 1.16 *p* = .71), with the nonverbal composite accounting for 18.6% of the variance in expressive language (*F*(6,331) = 21.27, *p* < .001; beta = .59; SE = .064 *p* < .001).

### Examination of the relationship between ASD-related social features and language in IBIS

An important issue for genetic discovery in ASD is the extent to which distinct genetic factors account for variation in ASD-related phenotypes. While the findings above support overlapping genetic factors for ASD and language delay, they do not address the extent of this overlap. In the case of high overlap, most of the same genetic factors would account for variation in language and social behavior, whereas in the case of lower partial overlap, some genes which contribute to ASD risk would exert independent effects on social and language phenotypes. We therefore tested whether the ADOS social affect score, an index of social performance quantifying ASD-related social deficits, influenced relationships between ASD risk and language scores. We reasoned that if the effect of ASD risk group on language was no longer significant when accounting for social affect score, then core ASD-related social symptoms and language function share a high degree of overlapping genetic factors. Alternatively, should the effect of ASD risk group on language persist when accounting for social affect score, then shared genetic contributors to autistic social features and language function would appear partially independent at this age.

A final step of hierarchical regression models is shown in Table 4. As above, models controlled for sex, maternal education, income, and race in the first step, followed by a second step adding risk group and a third step adding social affect score. Models including social affect score were significant for both receptive and expressive language (receptive *F*(7,327) = 9.65, *p* < .001; expressive *F*(7,327) = 7.27, *p* < .001), and social affect score contributed to variation in both receptive and expressive language. The contribution of ASD risk group to both receptive and expressive language remained significant with social affect score included (Table 4), consistent with partially independent genetic factors accounting for early ASD-related deficits in language and social behavior (see Additional file 1: Supplemental Results and Table S1, for consistent findings using the ADOS calibrated severity social affect scores).

**Table 4:** Group moderation of relationships between social performance and language

	Unstandardized coefficients		Standardized coefficients	*T*	Sig.	95.0% confidence interval for B	
	B	Std. error	Beta			Lower	Upper
**Receptive language model**							
(Constant)	53.90	2.85		18.90	< .001	48.29	59.51
**Sex**	3.21	1.05	.16	3.05	.002	1.14	5.28
Maternal education	2.23	1.27	.098	1.76	.080	− .27	4.73
**Income**	3.60	1.13	.18	3.19	.002	1.38	5.82
Race	− 2.61	1.54	− .087	− 1.70	.090	− 5.63	.41
**ASD risk status**	− 6.41	1.52	− .30	− 4.21	< .001	− 9.40	− 3.41
**Social affect**	− 1.35	.41	− .32	− 3.33	.001	− 2.14	− .55
Social affect by risk status	.92	.48	.21	1.92	.056	− .022	1.86
**Expressive language model**							
(Constant)	46.42	3.16		14.68	< .001	40.20	52.64
**Sex**	3.15	1.17	.14	2.70	.007	.86	5.45
**Maternal education**	4.06	1.41	.16	2.88	.004	1.29	6.82
**Income**	2.76	1.25	.12	2.21	.028	.30	5.22
Race	.16	1.70	.005	.096	.92	− 3.18	3.51
**ASD risk status**	− 6.41	1.69	− .27	− 3.80	< .001	− 9.73	− 3.094
**Social affect**	− 1.71	.45	− .37	− 3.80	< .001	− 2.59	− .82
**Social affect by risk status**	1.68	.53	.35	3.16	.002	.63	2.72

These parameters involve the fourth and final step in a hierarchical linear regression model in which either Mullen receptive or expressive language score is the dependent variable. Step 1 consists of the covariates sex, maternal education, and income; step 2 introduces autism spectrum disorder (ASD) risk status; and step 3 introduces social affect score on the Autism Diagnostic Observation Schedule, which measures autistic social deficits. The fourth step introduces an interaction term for ASD risk status and social affect. Bolded variables demonstrate a significant relationship with language. Social affect score is a significant contributor to variation in both receptive and expressive language. The interaction is significant for expressive language and shows a trend for receptive language. *std.* standard, *sig.* significance

The final step tested an interaction between ASD risk group and social affect score, as similar mean social affect scores for both LR-noASD and HR-noASD siblings (Table 1; see Additional file 1: Figure S2 for score distributions) implied a preservation of social performance relative to language in HR-noASD siblings. We therefore tested an interaction to determine whether the nature of a relationship between language and ASD-related social deficits differed between the risk groups. A significant interaction was found for expressive language (*p* = .002), as well as a trend-level interaction effect for receptive language (*p* = .056) (Table 4). Given these results, we next examined correlations for social affect and language scores separately for LR-noASD and HR-noASD groups. LR-noASD siblings showed moderate correlations between language and social affect scores (receptive: Spearman’s rho = − .33, *p* < .001.; expressive Spearman’s rho = − .41, *p* < .001), such that higher social affect scores, corresponding to greater ASD-related social deficits, were associated with lower language scores. In contrast, HR-noASD siblings exhibited no significant correlations between language and social affect scores (receptive: Spearman’s rho = − .069, *p* = .29; expressive: Spearman’s rho = − .051, *p* = .44; see Additional file 1: Supplemental Results, for similar findings when analyzing high-risk males and females separately). These differences in correlations for language and social affect scores were significant between LR-noASD and HR-noASD groups (receptive language: *Z* = − 2.33, *p* = .02; expressive language: *Z* = − 3.34, *p* < .001). By comparison, moderate correlations were present in both groups for language scores and nonverbal cognition, a developmental domain also related to language (LR-noASD receptive: Spearman’s rho = .62, *p* < .001; LR-noASD expressive: Spearman’s rho = .42, *p* < .001; HR-noASD receptive Spearman’s rho = .49, *p* < .001; HR-noASD expressive: Spearman’s rho = .53, *p* < .001).

## Discussion

### Convergent approaches support language endophenotypes of ASD

These findings demonstrate that decreased language function in early development, whether measured categorically as language delay or continuously as a dimensional language score, aggregates in unaffected toddlers at elevated genetic risk of ASD. The result was particularly robust for receptive language, which displayed an effect of ASD risk on language delay as well as continuous language scores. Analyses in IBIS also confirmed that the effect of ASD-risk status on language was significant when controlling for sociodemographic factors known to be associated with language. The positive associations observed between 24-month language and female sex, maternal education, and income, are in agreement with the existing literature, illustrating the representativeness of the IBIS sample.

The language differences observed in HR-noASD toddlers fulfill several previously elaborated criteria for endophenotypes. First, an IBIS sub-analysis showed lower indices of language function, a heritable ability [9], in high-risk siblings with ASD versus HR-noASD siblings, satisfying the criterion of co-segregation (i.e., increased co-inheritance) in affected family members. As stated above, lower language abilities also aggregated in HR-noASD siblings compared to LR-noASD siblings, with more frequent language delay and lower mean language scores reflecting a pathological shift in underlying score distributions across the HR-noASD group. These findings affirm that decrements in early language function are associated with increased genetic liability for ASD and represent ASD endophenotypes. Prior studies reporting enhanced signal detection of ASD-associated genetic variants when language is incorporated in phenotyping [87, 88] provide empirical support for language’s value added as an ASD endophenotype and for the ability of family studies to inform genetic investigations of ASD.

Our approach attempted to maximize detectable variation in language by focusing on a narrow age range characterized by rapid growth in foundational language skills. Our findings thus also suggest that genetic factors influencing early language development overlap with genetic risk for ASD. These results extend prior work from large family [89] and factor analytic studies [90, 91] linking language impairment in older children and adults with the occurrence of ASD. They also corroborate a recent meta-analysis showing lower language scores in high-risk toddler siblings, which included siblings with and without ASD [92]. We chose to present both the current meta-analysis and IBIS analyses here given that (1) this meta-analysis evaluated risk group differences involving not only language scores but also language delay, (2) the replication provided here confirms language differences in high-risk siblings even when restricting analyses to high-risk siblings *without* ASD, (3) the meta-analysis motivated more in-depth analyses in IBIS, and (4) the dual approaches attempted to address longstanding inconsistencies in the literature by providing more representative evidence for early language as an ASD endophenotype.

### Receptive language appears more affected than expressive language in high-risk siblings

HR-noASD siblings were three to four times more likely to exhibit language delay than LR-noASD siblings, consistent with a prior retrospective report [21], and IBIS analyses showed that receptive language delay, but not expressive language delay, accounts for much of this difference. Discrepant findings for receptive and expressive language delay are consistent with a lower signal for the expressive language endophenotype, which could be detected based on continuous scores but not the more stringent and less sensitive categorical language delay variable. Given that early language delay may be associated with subsequent language deficits [10, 47] and that receptive language impairment in particular is associated with worse functional outcomes than expressive language impairment [93], developmental surveillance in high-risk siblings with language delays may be especially important to capitalize on opportunities for intervention [94].

Summary effect sizes from the meta-analysis were also consistent with a larger effect of ASD-risk status for receptive language, and confidence intervals for receptive and expressive language were almost entirely non-overlapping (Fig. 1). This pattern parallels the preponderance of greater receptive versus expressive language deficits reported in children with ASD [95–98], as anticipated for an ASD endophenotype. Previous work has suggested that this discrepancy could reflect atypical mechanisms of language acquisition in ASD, whereby, in contrast to typical development, word comprehension appears less advanced than word production [97, 98]. While underlying mechanisms for this profile remain a question for future research, our findings reveal that at the group level, an analogous discrepancy in receptive and expressive language occurs in the context of ASD risk alone, without the elevated social deficits characteristic of ASD. These results also imply that the specific aspect of language measured may affect the ability to ascertain genetic associations between ASD and language and that some of the inconsistency in the literature could be resolved by distinguishing receptive and expressive language abilities.

One additional consideration regarding the distinct effects for receptive versus expressive language is the context of the assessment. Because the MSEL involves an interaction between an examiner and a child, the child’s social responsiveness, in itself a potential indicator of genetic liability for ASD, could impact his/her performance. It is possible that the evaluation of receptive language, which involves gauging a child’s response to an examiner’s prompt, may be more influenced by a child’s social responsiveness than expressive language, which may entail more self-motivated language output. While analyses in IBIS mitigate this concern by showing a similar relationship of ADOS social affect scores to both receptive and expressive language (Table 4), the persistent effect for ASD risk group in these models also implies the existence of shared genetic risk factors for ASD and language which are not associated with social performance. Thus, language assessments that reduce embedded social demands, for example, by using a psychophysiological measure such as auditory ERP to index language ability, rather than an interpersonal response, could be important for refining informative language endophenotypes of ASD.

### Specificity of the language endophenotype differs for receptive and expressive language

To explore the specificity of language endophenotypes, we tested the impact of nonverbal cognitive development on the relationship between risk status and quantitative language function. This point is of particular interest in the high-risk group, since a higher frequency of general cognitive deficits has been observed in family members of individuals with ASD [28, 89], and slightly lower mean cognitive scores were observed in IBIS and other infant sibling samples [99]. As expected, nonverbal cognition did contribute to variation in receptive and expressive language. For receptive language, a significant effect of risk status persisted even with the addition of nonverbal cognition to the model, indicating that the effect of risk status on receptive language is relatively specific. For expressive language, however, the effect of ASD-risk status was no longer significant when accounting for nonverbal cognition, in line with previously described interrelationships between general cognitive abilities and language [84–86]. These findings further support separating receptive and expressive language in behavioral genetic analyses and imply that therapies targeting domain-general abilities may improve language in children with genetic liability for ASD, as shown in emerging work for several populations with language impairment [100–103].

### Early social and language abilities are dissociated in high-risk toddlers without ASD

Because disrupted social and language development co-occur in ASD, we evaluated the relationship between language and autistic social deficits, based on the social affect score on the ADOS. As mentioned previously, the contribution of ASD risk status to both receptive and expressive language remained significant even when including social affect scores in the regression models. This suggests that heritable factors influencing early language and core autistic social features in the HR-noASD group are partially independent and that language deficits are unlikely to be purely secondary to ASD-related social deficits.

These analyses additionally revealed an interaction between risk group and social affect score, in which LR-noASD and HR-noASD siblings exhibited distinct relationships between language and social deficits. First, LR-noASD siblings displayed a negative relationship between levels of ASD-related social deficits and language. This concurred with a previously reported negative correlation between autistic traits and language in a general population toddler sample [104], confirming that the social affect score captured adequate variation in the low-risk group. In contrast, HR-noASD siblings showed no correlation between language and social domains. Like LR-noASD siblings, however, they showed moderate correlations between language and nonverbal cognition, reducing the likelihood that the dissociation of language and social domains was an epiphenomenon of broadly altered development.

A dissociation of early social and language abilities is reminiscent of the phenotype for the prior diagnosis of Asperger’s Disorder—in that case, early language development was relatively intact in the presence of autistic social impairment. At the same time, recent studies of older high-risk individuals without ASD have revealed an association of greater social deficits with lower language competency [35, 105]. In sum, while our findings show that genetic liability for ASD tracks with early language deficits, heritable factors for language and autistic social symptoms appear partially independent, and these phenotypes may not show steady covariation throughout development in HR-noASD siblings. Possible explanations include that some language-related social abilities are not measured by the ADOS, that language deficits may precede some ASD-related social deficits, or that, as has been described for specific language impairment, longstanding limitations in communication could reify a relationship between language and social function over time [106]. Although language impairment is no longer considered a core diagnostic feature of ASD in DSM-5 [107], behavioral genetic studies of trajectories of language development in conjunction with social development remain important to maximize the discovery of genetic contributors to ASD.

### Limitations

While these findings support ASD-related language endophenotypes at an early and prolific stage of language development, we note that associating language differences in HR-noASD siblings with specific genetic risk factors is required for direct confirmation of these endophenotypes. The results are also cross-sectional and the relationship of these early language differences to later outcomes warrant future study. Other work has reported quantitative language differences in high-risk infants [83], but studies of older HR-noASD siblings are mixed regarding the stability of language differences [22, 28, 108, 109], and further investigation is needed to determine the continuity of language deficits in this group. Resolution of language deficits in HR-noASD siblings would suggest that manifestations of genetic liability for ASD could occur transiently as part of a developmental process like language acquisition, consistent with the possibility of “state-dependent” developmental endophenotypes.

Although ASD diagnoses are generally stable by age 24 months [54–57], with recent work showing 82.3% positive predictive value from 24 to 36 months [110], there is also variation in the early development of ASD [110–112], which could affect the composition of the HR-noASD group and consequently, the magnitude of risk-related differences. We additionally acknowledge that in keeping with the infant sibling study design, control groups were comprised of low-risk participants, who in some cases exhibited relatively elevated mean standardized language scores, maternal education, and socioeconomic status. Similar characteristics were also observed in some of the HR-noASD samples as well, suggesting possible enrollment bias, as frequently occurs in studies requiring high participant engagement. However, this design allowed analyses of extensive developmental assessments generally unavailable in non-clinical samples, and the similar prevalence of language delay for pooled LR-noASD siblings and the general population, 5.2% versus 6% [113], supports the comparability of LR-noASD siblings. Finally, larger, more diverse samples than those presented here would be necessary to address potential heterogeneity and subtyping (e.g., simplex versus multiplex families); nevertheless, it is notable that the signal for decreased language function in HR-noASD siblings was appreciable in spite of this known heterogeneity.

## Conclusions

The detection of increased language delay in HR-noASD toddlers, both in existing literature and IBIS data, highlights the capacity to reliably measure heritable, clinically relevant markers of ASD risk during early development. At a practical level, the consistency of the findings across samples supports the utility of incorporating language metrics into early assessments, particularly for children at familial risk of ASD. Further characterization of the nature and continuity of language deficits in larger samples of ASD probands and their unaffected siblings is warranted (1) to refine genetically informative language phenotypes, taking into account receptive and expressive language, as well as potential contributions of social responsiveness, and (2) to resolve how deviations in early language development correspond to later ASD-related outcomes, e.g., pragmatic language deficits, which also occur more frequently in HR-noASD siblings [114]. Such studies would provide a valuable opportunity to clarify the heterogeneity of the genetic architecture ASD as well as the role and timing of language-based interventions.

## Additional file

Additional file 1: **Table S1.** Study Characteristics of Publications in Secondary Meta-analysis. **Table S2.** Group Moderation of Relationships between Social Performance and Language Using ADOS Calibrated Severity Social Affect Scores. **Figure S1.** Secondary Meta-analysis of Language Scores in High-risk Siblings without ASD. **Figure S2.** Social Affect Scores at 24 months of Age in IBIS Infant Siblings. (DOCX 224 kb)

## Abbreviations

ADOS: Autism Diagnostic Observation Schedule
ASD: Autism spectrum disorder
BSRC: Baby Siblings Research Consortium
CNTNAP: Contactin-associated protein
DSM: Diagnostic and Statistical Manual of Psychiatric Disorders
HR-noASD: High-risk siblings without autism spectrum disorder
IBIS: Infant Brain Imaging Study
LR-noASD: Low-risk siblings without autism spectrum disorder
MRI: Magnetic resonance imaging
MSEL: Mullen Scales of Early Learning
NOS: Not otherwise specified
