# Supplementary material for: Language delay aggregates in toddler siblings of children with autism spectrum disorder
Source: J Neurodev Disord. 2018 Oct 22;10:29. doi: 10.1186/s11689-018-9247-8 (PMC6198516; doi:10.1186/s11689-018-9247-8)
Supplement: Supplementary file 1 — Table S1. Study Characteristics of Publications in Secondary Meta-analysis. Table S2. Group Moderation of Relationships between Social Performance and Language Using ADOS Calibrated Severity Social Affect Scores. Figure S1. Secondary Meta-analysis of Language Scores in High-risk Siblings without ASD. Figure S2. Social Affect Scores at 24 months of Age in IBIS Infant Siblings. (DOCX 224 kb) [file 11689_2018_9247_MOESM1_ESM.docx]

**Supplemental Results**

Confirmation of Decreased Language Abilities for High-risk Siblings with ASD

Work from multiple infant sibling samples has shown decreased levels of language in high-risk siblings with ASD (HR-ASD siblings) versus high-risk siblings without ASD (HR-noASD siblings) [1-3]. To formally test in the IBIS sample whether high-risk siblings with ASD (HR-ASD siblings) show lower language abilities than HR-noASD siblings, a criterion for an endophenotype, we performed a sub-analysis comparing HR-ASD siblings, who were not included in the primary analyses (n=61), to HR-noASD siblings. In keeping with the anticipated risk-group relationships for an endophenotype, the ASD-affected group showed a greater prevalence of language delay, 60.7% (*χ*^2^(1)=59.94, *p*<.001), than HR-noASD siblings, with 13.6% language delay. Examining receptive and expressive language delay separately revealed similar differences, with 55.7% of HR-ASD siblings having receptive language delay versus 9.4% of HR-noASD siblings (*χ*^2^(1)=67.90, *p*<.001) and 32.8% of HR-ASD siblings having expressive language delay versus 7.7% of HR-noASD siblings (*χ*^2^(1)=27.33, *p*<.001).

Similarly, HR-ASD siblings displayed lower mean receptive and expressive language scores than the HR-noASD group (Receptive language: HR-ASD=35.87(14.89) versus HR-noASD=51.79(10.43), *t*(76)=-7.87, *p*<.001, Cohen’s *d*=1.24; Expressive language: HR-ASD=38.21(11.50), HR-noASD=49.13(11.29), expressive: *t*(294)=-6.70, *p*<.001, Cohen’s *d*=0.96). By both categorical and continuous language measures, a greater prevalence of decreased language function was observed in HR-ASD siblings in comparison to HR-noASD siblings in IBIS, consistent with the co-segregation criterion of an endophenotype in affected versus unaffected family members. Additionally, the qualitative profile of these language differences, whereby receptive language appears more strongly affected in HR-ASD siblings than expressive language, corresponds both to the published observations for toddlers with ASD [4,5], as well as findings in the main text that receptive language appears more affected than expressive language in HR-noASD siblings (Figure 1). The consistency of this profile at differing levels of ASD risk further supports an association between familial risk factors for ASD and language function in early development.

Correlations of ADOS Raw Social Affect Scores and Risk Group by Sex

We explored possible sex-specific differences in the relationship between language and raw ADOS social affect score, given known sex differences in language development [6,7] and prevalence of ASD. The lack of a correlation for HR-noASD siblings was maintained when analyzing HR-noASD males and females separately (females: n=100; receptive language: r=-.035, *p*=.71; expressive language: r=.095, *p*=.35; males: n=132; receptive language: r=-.15, *p*=.089; expressive language: r=-.11, *p*=.21).

**Table S1.** Study Characteristics of Publications in Secondary Meta-analysis

| Publication | HR-/LR- | Mean Age  (mo.) | Language Delay Criteria | Clinical Best Estimate Measures | Language Delay  (HR versus LR) | MSEL  Receptive Language | MSEL Expressive Language | |
| --- | --- | --- | --- | --- | --- | --- | --- | --- |
| Mitchell et al., 2006 [3] | 91/52 | 24 | ≤1.5 SD mean: MSEL or PLS | ADOS  DSM-IV-TR | OR=1.65  (.50-5.47)  Χ^2^=.29, p=.59 | **118.03 (12.72) 106.09 (18.42)** | **106.98 (19.39)**  **103.22 (16.10)** | |
| Toth et al., 2007 [8] | 42/20 | 21 | n/a | ADOS  ADI-R  DSM-IV-TR | n/a | **54.50 (6.40)**  **46.55 (15.60)** | 48.45 (6.65)  49.07 (12.24) | |
| Stone et al., 2007 [9] | 64/42 | 16 | n/a | n/a | n/a | 48.2 (10.8)  43.8 (12.6) | 48.7 (10.5)  45.1 (11.1) | |
| Paul et al., 2011 [10] | 38/31 | 12 | n/a | n/a | n/a | **46.1 (7.2)**  **41.1 (9.7)** | **47.4 (12.0)**  **40.8 (10.7)** | |
| Curtin & Vouloumanos, 2013 [11] | 38/31 | 12 | n/a | n/a | n/a | **45.97 (7.11)**  **42.16 (7.14)** | 53.93 (10.47)  49.87 (11.04) | |
| Ozonoff et al., 2014 [12] | 243/116 | 24 | n/a | ADOS  DSM-IV-TR | n/a | **25.6 (3.6)**  **23.2 (3.5)** | **23.4 (2.7)**  **22.1 (2.7)** | |
| Finch et al., 2017 [13] | 61/71 | 24 | n/a | ADOS | n/a | **58.82 (7.8)**  **54.55 (8.9)** | **56.65 (9.8)**  **53.41 (9.9)** | |
| This meta-analysis of language scores replaces the large Baby Sibling Research Consortium (BSRC) Study by Messinger et al. [2] with smaller studies [3, 12, 13] whose subjects overlapped with the BSRC study. Except where stated, language delay was defined as either receptive or expressive language delay. Odds ratios (ORs) for language delay are listed with 95% confidence intervals in parentheses. Language scores consist of T-scores on the Mullen Scales of Early Learning (MSEL) for all studies except Mitchell et al. [3] and Ozonoff et al [12]. The Mitchell study uses MSEL standard scores (available in 81 HR siblings and 46 LR siblings) while the Ozonoff study uses estimates of raw MSEL scores controlled for age, sex, study site, and funding phase from a mixed effects linear model. Scores for LR-noASD siblings are listed first and HR-noASD siblings are listed second. Bolded values are significant at *p*<.05. mo.=months. HR=high-risk siblings without ASD. LR=low-risk siblings without ASD. ASD=Autism Spectrum Disorder. M-CDI=MacArthur Communicative Development Inventory. PLS=Preschool Language Scale. ADOS=Autism Diagnostic Observation Schedule. ADOS-G=Autism Diagnostic Observation Schedule-General. ADI-R=Autism Diagnostic Interview-Revised. DSM-IV-TR=Diagnostic and Statistical Manual of Psychiatric Disorders, 4^th^ edition, text revision. | | | | | | | |  |

**Table S2.** Group Moderation of Relationships between Social Performance and Language Using ADOS Calibrated Severity Social Affect Scores

| **Receptive Language**  **Model** | Unstandardized Coefficients | | | | Standardized Coefficients | | T | | Sig. | 95.0% Confidence Interval for B | | |
| --- | --- | --- | --- | --- | --- | --- | --- | --- | --- | --- | --- | --- |
|  | B | | Std. Error | | Beta | |  |  |  | Lower | Upper | |
| (Constant) | 54.91 | | 3.14 | |  | | 17.49 | | <.001 | 48.73 | 61.083 | |
| **Sex** | 3.355 | | 1.062 | | .16 | | 3.16 | | .002 | 1.26 | 5.44 | |
| Maternal Education | 2.15 | | 1.28 | | .094 | | 1.68 | | .095 | -.38 | 4.67 | |
| **Income** | 3.54 | | 1.14 | | .17 | | 3.098 | | .002 | 1.29 | 5.79 | |
| Race | -2.67 | | 1.55 | | -.089 | | -1.72 | | .086 | -5.72 | .38 | |
| **ASD Risk Status** | -7.67 | | 2.11 | | -.36 | | -3.65 | | .000 | -11.81 | -3.53 | |
| **Calibrated Social Affect** | -2.18 | | .85 | | -.25 | | -2.57 | | .011 | -3.86 | -.51 | |
| Social Affect by Risk Status | 1.75 | | 1.006 | | .22 | | 1.74 | | .083 | -.23 | 3.73 | |
|  | | | | | | | | | | | | |
| **Expressive Language**  **Model** | Unstandardized Coefficients | | | Standardized Coefficients | | T | | Sig. | | 95.0% Confidence Interval for B | | |
|  | B | Std. Error | | B | |  |  |  |  | Lower | | Upper |
| (Constant) | 47.66 | 3.47 | |  | | 13.72 | | <.001 | | 40.83 | | 54.50 |
| **Sex** | 3.32 | 1.18 | | .15 | | 2.83 | | .005 | | 1.008 | | 5.63 |
| **Maternal Education** | 4.053 | 1.42 | | .16 | | 2.86 | | .005 | | 1.26 | | 6.84 |
| **Income** | 2.55 | 1.26 | | .11 | | 2.016 | | .045 | | .062 | | 5.036 |
| Race | .048 | 1.72 | | .001 | | .028 | | .98 | | -3.33 | | 3.42 |
| **ASD Risk Status** | -8.65 | 2.33 | | -.37 | | -3.71 | | <.001 | | -13.23 | | -4.066 |
| **Calibrated Social Affect** | -2.71 | .94 | | -.28 | | -2.88 | | .004 | | -4.57 | | -.86 |
| **Social Affect by Risk Status** | 3.24 | 1.11 | | .38 | | 2.91 | | .004 | | 1.05 | | 5.43 |
| These parameters involve the fourth and final step in a hierarchical linear regression model in which either Mullen receptive or expressive language score is the dependent variable. Step 1 consists of the covariates sex, maternal education, income, and race; step 2 introduces autism spectrum disorder (ASD) risk status; and step 3 introduces the calibrated severity social affect score on the Autism Diagnostic Observation Schedule [14], which measures autistic social deficits. The fourth step introduces an interaction term for ASD risk status and calibrated severity social affect score. Bolded variables demonstrate a significant relationship with language. Calibrated social affect score is a significant contributor to variation in both receptive and expressive language. The interaction is significant for expressive language and shows a trend for receptive language. | | | | | | | | | | | | |


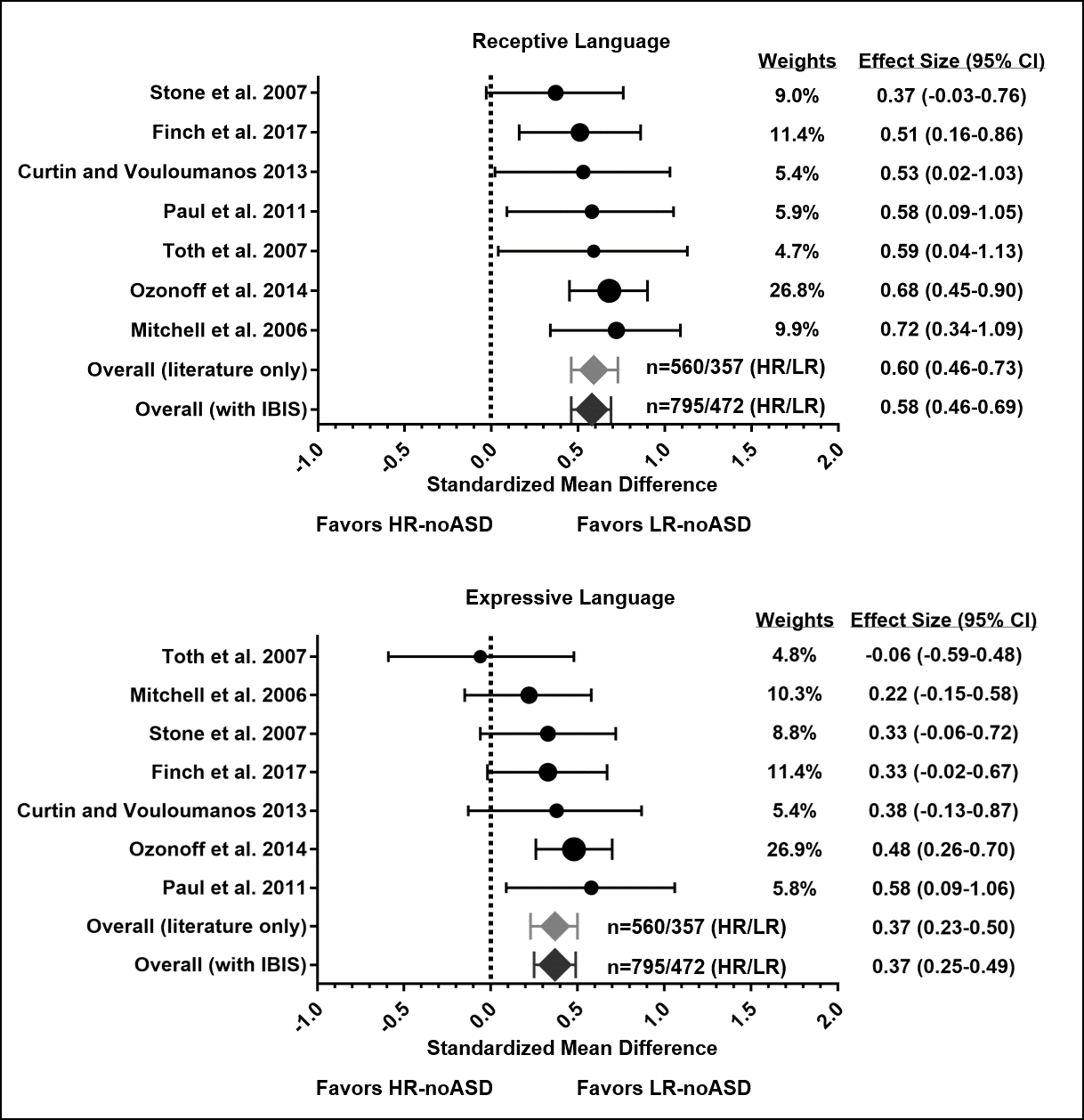


**Figure S1.** Secondary Meta-analysis of Language Scores in High-risk Siblings without ASD

Forest plots illustrate results of the meta-analysis examining differences in receptive and expressive language scores between low-risk siblings without ASD (LR-noASD) and high-risk siblings without ASD (HR-noASD). Circle sizes illustrate each study’s weighted impact when including IBIS data, with values for weights and effect sizes listed on the right. Error bars represent 95% confidence intervals (CI). Summary weighted effect sizes for published studies, indicated as ‘Overall (literature only),’ are shown as a light gray diamond; the dark gray diamonds show the result including IBIS data. Numbers of subjects in HR-noASD (HR) and LR-noASD (LR) groups are adjacent to these diamond markers. Both summary effect sizes (i.e., standardized mean differences) indicate that receptive and expressive language scores are significantly lower in high-risk siblings. The effect size is moderate for receptive language and small for expressive language.


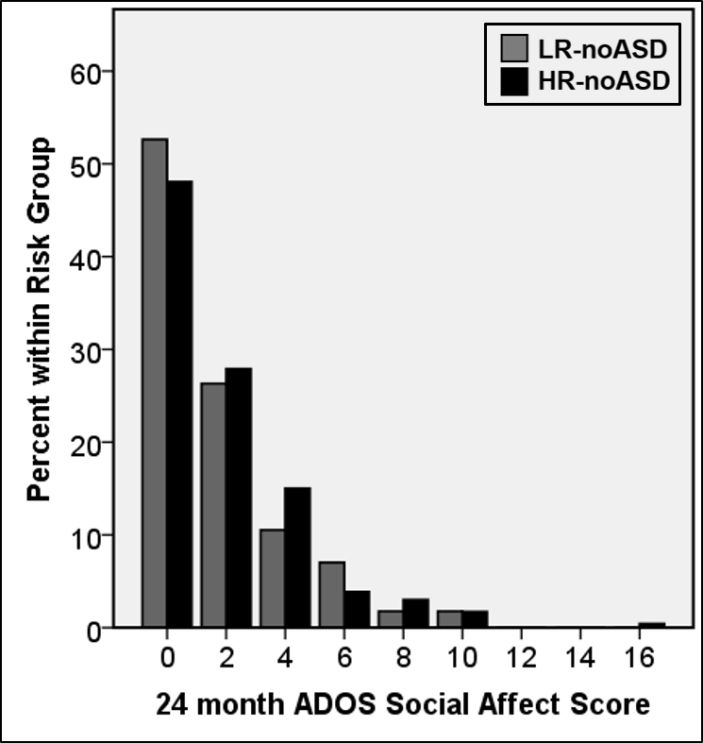


**Figure S2.** Social Affect Scores at 24 months of Age in IBIS Infant Siblings

A histogram for social affect scores (based on the summed item-level values for the social affect factor described by Gotham and colleagues [15] on the Autism Diagnostic Observation Schedule (ADOS) at 24 months of age shows overlapping distributions across a similar range for unaffected siblings at low familial risk of ASD (LR-noASD, gray bars) and high familial risk of ASD (HR-noASD, black bars). Significant results do not change when the high-scoring subject in the HR-noASD group is removed from analyses.

**REFERENCES**

1. Landa, RJ, Stuart, EA, Gross, AL, Faherty, A. Developmental Trajectories in Children With and Without Autism Spectrum Disorders: The First 3 Years. Child Development. 2013;84:429–442.

2. Messinger DS, Young GS, Webb SJ, Ozonoff S, Bryson SE, Carter A, et al. Early sex differences are not autism-specific: A Baby Siblings Research Consortium (BSRC) study. Mol Autism. 2015; 6:32.

3. Mitchell S, Brian J, Zwaigenbaum L, Roberts W, Szatmari P, Smith I, et al. Early language and communication development of infants later diagnosed with autism spectrum disorder. J Dev Behav Pediatr. 2006;27:S69-78

4. Charman T, Drew A, Baird C, Baird G. Measuring early language development in preschool children with autism spectrum disorder using the MacArthur Communicative Development Inventory (Infant Form). Journal of Child Language. 2003;30:213-36.

5. Hudry K, Leadbitter K, Temple K, Slonims V, McConachie H, Aldred C, Howlin P, Charman T, Pact Consortium. Preschoolers with autism show greater impairment in receptive compared with expressive language abilities. International Journal of Language & Communication Disorders. 2010;45:681-90.

6. Fenson L, Dale PS, Reznick JS, Bates E, Thal DJ, Pethick SJ. Variability in early communicative development. Monogr Soc Res Child Dev. 1994;59:1-173; discussion 174-85.

7. Huttenlocher J, Haight W, Bryk A, Seltzer M, Lyons T. Early Vocabulary Growth: Relation to Language Input and Sex. Dev Psychol. 1991;27:236-48.

8. Toth K, Dawson G, Meltzoff AN, Greenson J, Fein D. Early social, imitation, play, and language abilities of young non-autistic siblings of children with autism. J Autism Dev Disord. 2007;37:145-57.

9. Stone WL, McMahon CR, Yoder PJ, Walden TA. Early social-communicative and cognitive development of younger siblings of children with autism spectrum disorders. Arch Pediatr Adolesc Med. 2007;161:384-90.

10. Paul R, Fuerst Y, Ramsay G, Chawarska K, Klin A. Out of the mouths of babes: vocal production in infant siblings of children with ASD. J Child Psychol Psychiatry. 2011;52:588-98.

11. Curtin S, Vouloumanos A. Speech preference is associated with autistic-like behavior in 18-months-olds at risk for Autism Spectrum Disorder. J Autism Dev Disord. 2013;43:2114-20.

12. Ozonoff S, Young GS, Belding A, Hill M, Hill A, Hutman T, et al. The Broader Autism Phenotype in Infancy: When Does It Emerge? Journal of the American Academy of Child & Adolescent Psychiatry. 2014;53:398-407.

13. Finch KH, Seery AM, Talbott MR, Nelson CA, Tager-Flusberg H. Lateralization of ERPs to speech and handedness in the early development of autism spectrum disorder. J Neurodev Disord. 2017;9:4.

14. Hus V, Gotham K, Lord C. Standardizing ADOS domain scores: Separating severity of social affect and restricted and repetitive behaviors. Journal of Autism and Developmental Disorders. 2014;44:2400-12.

15. Gotham K, Risi S, Pickles A, Lord C. The Autism Diagnostic Observation Schedule: revised algorithms for improved diagnostic validity. J Autism Dev Disord. 2007;37:613-27.
